# The connection between mindfulness levels and life satisfaction among breast cancer patients: the sequential mediating effects of body appreciation and stress perception

**DOI:** 10.7717/peerj.20485

**Published:** 2025-12-18

**Authors:** Junli Zhou, Xianhui Huang, Dan Yuan, Liyuan Wang, Minyu Liu, Xue Jiang, Lijuan Xing, Song Xu

**Affiliations:** 1 Department of Psychology, The 991st Hospital of the Chinese PLA, Xiangyang, China; 2 Xiangyang Women’s Federation, Xiangyang, China; 3 Gynecology, The 991st Hospital of the Chinese PLA, Xiangyang, China

**Keywords:** Mindfulness, Body appreciation, Perceived stress, Life satisfaction, Breast cancer, Chain mediation

## Abstract

**Background:**

Research indicates a positive relationship between life satisfaction, mindfulness, and emotional perception. While this connection is observed in a variety of groups, it has been less studied in women with breast cancer. The research investigated correlation among mindfulness, body appreciation, emotional perception, and life satisfaction in Chinese breast cancer patients.

**Methods:**

In Xiangyang City, a survey was conducted involving 312 individuals diagnosed with breast cancer. The instruments used for this assessment included the Revised Mindfulness Cognition and Affective Scale (CAMS-R), Body Appreciation Scale (BAS), Perceived Stress Scale (PSS), and Life Satisfaction Scale (SWLS).

**Results:**

(1) Mindfulness may positively and substantially predict the life satisfaction of breast cancer patients (*β* = 0.439, *P* < 0.001); (2) body appreciation (*β* = 0.152, *P* < 0.01) and perceived stress (*β* =  − 0.183, *P* < 0.01), respectively, acted as a mediator to the influence of being mindful on fulfillment in life, and (3) mindfulness could also indirectly affect the life satisfaction of individuals diagnosed with breast cancer through the sequential influence of body appreciation and perceived stress.

**Conclusion:**

The mediating model has been fitted on cross-sectional data in this research. Breast cancer patients’ life satisfaction is significantly positively predicted by mindfulness, which includes indirect as well as direct outcomes of perceived stress and body appreciation. The connection between mindfulness and overall well-being is partially mediated through body appreciation and also perceived stress, and the chain mediation effect provides additional insight into the connection between mindfulness and overall well-being. Therefore, in clinical practice, mindfulness training can enhance the body appreciation levels of breast cancer patients, reduce their perception of stress, and subsequently improve their overall life satisfaction.

## Introduction

At present, cancer treatment has entered the era of “precision strike”, and the long-term quality of life of cancer patients after cure is increasingly valued (Sonkin, Thomas & Teicher, 2024). Breast cancer is the most common of the 38 malignant tumors worldwide (Barzaman et al., 2020). According to statistics from the American Cancer Society, there were about 2.309 million cases of breast cancer worldwide in 2022, accounting for 11.6% of all female malignant tumors. The worldwide occurrence of breast cancer continues to rise annually, with projections indicating that by 2024, the yearly incidence will surpass three million cases (Bray et al., 2024; Yuan et al., 2024). Advancements in the early diagnosis of breast cancer and treatment methods like targeted hormones have led to improved survival outcomes (Katsura et al., 2022). As a matter of fact, a breast cancer diagnosis can be a serious physical, psychological and financial burden for patients and their families (Javan Biparva et al., 2024; Den & Sisti, 2023). Therefore, in addition to considering the patient’s disease prognosis, the life satisfaction of breast cancer patients is also an important outcome indicator. Symptoms and adverse reactions at various stages of the disease, along with negative emotions like anxiety, fear, and depression, as well as a diminished quality of life, are all significant factors that contribute to the reduction of life satisfaction in breast cancer patients (Ghose et al., 2024).

Breast cancer diagnosis can cause emotional distress in patients, which can affect subjective well-being. One crucial cognitive measure of subjective well-being is life satisfaction (Tokay Argan & Mersin, 2021). Life satisfaction is a key indicator of a person’s perceived well-being. As one of the core cognitive components of the Diener subjective well-being model, life satisfaction reflects the overall evaluation of breast cancer patients on treatment process, interpersonal support, self-worth and other life fields (Tokay Argan & Mersin, 2021; Srivastava et al., 2021). Evidence indicates that psychosocial issues can significantly amplify the severity of physical symptoms associated with diseases (Cieślak et al., 2022; Javan Biparva et al., 2023). Breast cancer patients, due to both physical and psychological trauma, frequently experience less life satisfaction. As females are the most important members of the family, their life satisfaction not only affects their survival but also the cohesion of their family structure (Soria-Reyes et al., 2023). Consequently, it is essential to emphasize the importance of individuals’ well-being after a breast cancer diagnosis, and a primary goal of breast cancer treatment should be to enhance patients’ life satisfaction. This research investigates the inherent elements that influence breast cancer patients’ life satisfaction, offering a guide for reducing their stress perception and improving their subjective well-being.

### Mindfulness, body appreciation, and life satisfaction

In positive psychology, mindfulness refers both to a state of non-judgmental awareness and to certain meditation practices (Lundh, 2024). Mindfulness is commonly defined as the intentional act of focusing on the present moment, free from judgment, while maintaining a curious and open mindset, which fosters awareness (Dahl & Davidson, 2019). The degree of mindfulness affects the individual’s indicators of well-being, such as sleep quality, mental resilience, life satisfaction, *etc.* (Zollars, Poirier & Pailden, 2019). For example, research on mindfulness has shown benefits in improving employee well-being and as a protective factor, with high mindfulness levels linked to greater life satisfaction and decreased emotional distress (MacAulay et al., 2022). Similarly, mindfulness plays a beneficial role in reducing stress and enhancing health (Telles et al., 2024). A thorough analysis carried out by Wang et al. (2024) demonstrated that mindfulness notably alleviated stress, anxiety, and distress in patients with breast cancer; small to moderate positive effects were observed for life satisfaction and positive emotions. Therefore, improving an individual’s level of mindfulness is to enhance one’s self-awareness, thereby improving bad mood and increasing life satisfaction.

Body image is how a person perceives, feels, and reacts to physical characteristics like weight, body shape, and appearance (Rhoten, 2016). Previous studies have concentrated on adverse aspects of body image or physical dissatisfaction (Linardon et al., 2022). With the rise of optimism in psychology, research on body image has transitioned from focusing on negative aspects and toward positive ones, with healthy body image regarded as a positive perception of the body. Measurement and cultivation of body appreciation is of great significance for maintaining women’s physical and mental health (Liang et al., 2021; Mele, Cazzato & Urgesi, 2013). Body appreciation not only reduces negative emotions but also promotes overall health and well-being. Positive physical intentions are related to indicators of mental and physical health, including life satisfaction, subjective well-being, and optimism (Aimé et al., 2020; Tylka, 2018). Research indicates that individuals who express gratitude for their bodies are more inclined to adopt positive healthy behaviors (Tylka, 2018). Individuals with breast cancer frequently undergo significant bodily changes, which can affect how they view themselves and life satisfaction. A study on body image and happiness among breast cancer patients has revealed that women who appreciate their bodies and their functions experience greater happiness compared to those who are dissatisfied with their body function (Ettridge et al., 2022). Therefore, appreciating one’s body can promote overall well-being and increase satisfaction.

Similarly, mindfulness has been associated with improvements in positive body image. Current evidence shows that mindfulness positively influences body image, and engaging in mindfulness practices can enhance body appreciation, self-esteem, and mood (Chang, 2025). A study of 376 women found that yoga practice promoted improved the female participants ’mindfulness levels, which in turn led to a greater appreciation of the body (Cox & McMahon, 2019). Balciuniene showed that female undergraduates experienced an improvement in body appreciation scores after a brief mindfulness body scan (gradually focusing on body sensations from feet to head) (Balciuniene, Jankauskiene & Baceviciene, 2022). It can be seen that body appreciation moderates the connection between life satisfaction and mindfulness. Therefore, we proposed the following hypothesis:

H1: Body appreciation mediates between life satisfaction and mindfulness.

### Mindfulness, perceived stress, and life satisfaction

Perceived stress refers to the degree to which an individual feels stress regarding themselves following an event, as well as their physical and psychological responses to it (Heruti, Levy & Avitsur, 2018). An individual’s perception of stress depends on personality, learning, and culture (Heinrich & O’Connell, 2024). Breast cancer patients often experience higher levels of perceived stress due to the pain of the disease and various adverse effects in treatment (Vafaei et al., 2023). Zollars, Poirier & Pailden (2019) discovered that mindfulness was inversely related to anxiety, depression, and perceived stress. Research shows that an individual’s level of mindfulness is a significant negative predictor of stress; in other words, greater mindfulness can lessen stress perception and mitigate the adverse effects of negativity (Berdida, Lopez & Grande, 2023; Chen et al., 2022; Hilcove et al., 2021). Other studies have shown that individuals with higher mindfulness can reduce psychological stress and its negative effects and improve individual quality of life and subjective well-being (Lightsey Jr & Smith IV, 2024; Schulte-Frankenfeld & Trautwein, 2022). Individual mindfulness, as an intrinsic resource for coping with stress, is a protective factor in stressful situations (Hoover et al., 2022). The individual level of mindfulness in breast cancer patients affects their perceived stress and psychological distress, which in turn affects the life satisfaction of breast cancer patients. A growing body of research has found that practicing mindfulness can relieve stress, regulate mood, and improve emotional state (Knoerl et al., 2022). A systematic review showed that mindfulness-based therapy can reduce stress and improve life satisfaction in people with breast cancer (Haller et al., 2017). Thus, the following hypothesis were suggested:

H2: Perceived stress can serve as a mediator in the connection between mindfulness and life satisfaction.

### Mindfulness, body appreciation, perceived stress, and life satisfaction

By promoting the enhancement in the mindfulness level of individuals with breast cancer, it can bring high life satisfaction, and breast cancer patients can achieve a positive body image by improving their mindfulness perception ability, thereby enhancing the subjective state of mind of individuals after being diagnosed with breast cancer (Ettridge et al., 2022). Research has found that body appreciation is negatively correlated with perceived stress. Positive body appreciation can maintain mental health and reduce personal stress (Rosenbaum, Gillen & Bloomer, 2024; Shi et al., 2024). Therefore, the following hypothesis were proposed:

H3: Body appreciation and perceived stress will act as mediators in the relationship between mindfulness and life well-being in individuals with breast cancer.

**Figure 1:**
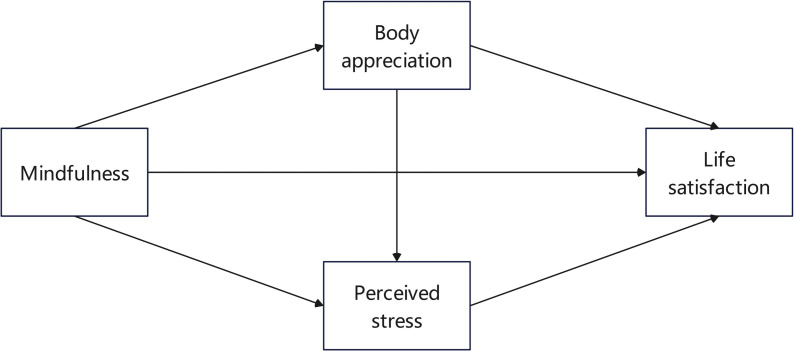
The hypothetical relationship model.

While some research has examined the effects of mindfulness on life satisfaction among individuals with breast cancer, there is a scarcity of studies investigating the roles of positive body imagery and perceived stress, particularly in women facing this disease (Marco-Salvador et al., 2024). A breast cancer diagnosis can adversely affect both the physical health and psychological well-being of the patient (Javan Biparva et al., 2023). This investigation focuses on breast cancer patients as research subjects and thoroughly explores the internal structure of how mindfulness impacts life satisfaction. The findings aim to enhance studies on the well-being of individuals with breast cancer and offer recommendations for improving their life satisfaction. Figure 1 presents the proposed conceptual model of the study.

## Resources and Procedures

### Participants

This research involved 312 women who had been affected by breast cancer that were treated at four hospitals in Xiangyang City between October 2023 and November 2024. Patients with any comorbidities or psychiatric conditions did not participate in the study. Questionnaires were distributed to patients only after obtaining their informed consent. The study protocol had been approved and met the ethical criteria set by the Institutional Ethics Committee of the 991st Hospital of the Chinese People’s Liberation Army (YJ-991[2024]14).

### Measurements

#### Mindfulness

Feldman et al. (2007) developed the CAMS-R, which is commonly employed to evaluate mindfulness tendencies. The CAMS-R utilized in this investigation was revised by Chan et al. (2016) at Shue Yan University in Hong Kong. The rating scale consists of twelve variables and is assessed on a 4-point Likert scale, with elevated scores indicating greater degrees of mindfulness. The Cronbach’s alpha coefficient for the scale in this study was 0.939.

#### Body appreciation

The Body Appreciation Scale (BAS) developed by Avalos, Tylka & Wood-Barcalow (2005) was utilized to assess individuals’ positive body image. The rating system is graded on a 5-point Likert scale using an aggregate consisting of ten components, with elevated scores reflecting a greater level of body appreciation. In this study, the Cronbach’s alpha coefficient for this scale was 0.926.

#### Perceived stress

Cohen, Kamarch & Mermelstein (1983) created the Chinese version of the PSS, which was utilized to assess an individual’s perceived stress. The scale utilizes a 5-point Likert scale comprising 14 items in total. A higher cumulative score indicates a higher perceived level of stress. The Cronbach’s alpha coefficient for this scale in this study was 0.94.

#### Life Satisfaction

Diener et al. (1985) developed the SWLS, a tool widely used to assess an individual’s subjective perception of their quality of life and to measure their overall level of life satisfaction. The scale consists of five items and is scored by Likert 7-scale. The Cronbach’s alpha coefficient for this scale in this research was 0.896.

### Procedure

This study explored the relationship between mindfulness and life satisfaction in breast cancer patients by cross-sectional survey. The period of data collection was October 2023–November 2024. Breast cancer patients were recruited from four hospitals in Xiangyang City. Participants were requested to fill out the questionnaire online using a mobile phone upon reading the details on the form and giving their consent in writing. Instruct participants who don’t use smartphones to fill out paper questionnaires.

Participation in this study is entirely voluntary, and all personal data will be maintained in strict confidentiality. All procedures employed in this research adhered to the 1964 Helsinki Declaration, and the Ethics Committee of the 991st Hospital of China People’s Liberation Army has granted approval for this study (No. YJ-991[2024]14).

### Data analysis

Using Harman’s one-factor model, the common method variance (CMV) problem was confirmed (Beasley, 2014). Descriptive statistics were conducted using SPSS version 27.0. Mean and standard deviation were calculated for continuous variables, while frequency and percentage were determined for categorical variables. Bias testing and correlation analysis were performed using standard procedures. The mediating effect was assessed using Hayes’ SPSS Macro program Model 6, incorporating a 95% confidence interval and Bootstrap bias correction test. The model’s overall, direct, and indirect effects were evaluated, and if the 95 percent bootstrap confidence interval did not include zero, the intermediary effect was deemed significant (Beasley, 2014).

## Outcomes

### Common methods bias test

The standard technique bias was assessed using Harman’s one-way test. All measurement items were subjected to exploratory factor examination. The findings showed that seven variables had eigenvalues exceeding one, with the variance explained by the first factor accounting for 32.71% of the total, which fell below the critical threshold of 40%. Thus, the impact of common method bias on the data was not significant.

### Sample characteristics

The survey initially comprised 312 breast cancer patients, all of whom were women, with an average age of 52.38 ± 8.04 years. 90.1% of the participants were married, while 88.5% had children. The average level of mindfulness was 28.78 ± 7.68, and the average level of life satisfaction was 43.55 ± 12.59. The marital status, residence status, education level and occupation of the participants are shown in Table 1.

### Pearson correlation analysis

According to Pearson correlation analysis, mindfulness had an inverse relationship with stress perception (*r* =  − 0.391, *P* < 0.01) and a direct relationship with life satisfaction and body appreciation (*r* = 0.352, *P* < 0.01; *r* = 0.439, *P* < 0.01). Life satisfaction and body appreciation had a positive correlation (*r* = 0.326, *P* < 0.01), while perceived stress and body appreciation had a negative correlation (*r* =  − 0.344, *P* < 0.01). Life satisfaction and perceived stress had a negative correlation (*r* =  − 0.359, *P* < 0.01). According to this research, life satisfaction in breast cancer patients rises with higher mindfulness and body appreciation and with lower perceived stress. Table 2 displays the findings.

**Table 1:** Demographic and clinical characteristics of participants (*N* = 312).

**Characteristics**	**Variables**	**N**	**N%**
Marital status	Unmarried	9	2.9
	Married	281	90.1
	Divorce	16	5.1
	Remarry	2	0.6
	Widowed	4	1.3
Whether you have children	Yes	276	88.5
	No	36	11.5
Residency status	Living alone	20	6.4
	Living with a spouse	55	17.6
	Living with children	34	10.9
	Living with your spouse and children	189	60.6
	Other	14	4.5
Educational level	Elementary school	1	0.3
	Junior high school	21	6.7
	High school	57	18.3
	Undergraduate	228	73.1
	Graduate student	5	1.6
Household income	<1,000 RMB	11	3.5
	1,000∼2,999 RMB	127	40.7
	3,000∼6,999 RMB	116	37.2
	7,000∼9,999 RMB	36	11.5
	≥10,000 RMB	22	7.1
Occupation	Farmer	34	10.9
	Worker	31	9.9
	Teacher	5	1.6
	Medical personnel	3	1
	Public servant	59	18.9
	Freelancing	27	8.7
	Unemployed	4	1.3
	Retire	3	1
	Other	146	46.8
Duration of illness (year)	<1 year	56	17.9
	1∼3 years	139	44.6
	>3 years	117	37.5

**Table 2:** Correlations among study variables (*N* = 312).

	Mean ± SD	SWLS	BAS	PSS	CAMSR
SWLS	4.31 ± 1.49	1	.326^**^	−.359^**^	.439^**^
BAS	5.05 ± 1.34	.326^**^	1	−.344^**^	.352^**^
PSS	3.11 ± 0.89	−.359^**^	−.344^**^	1	−.391^**^
CAMSR	2.39 ± 0.64	.439^**^	.352^**^	−.391^**^	1

**Notes.**

CAMSR Mindfulness BAS Body appreciation PSS Perceived stress SWLS Life satisfaction Mean ± SD means and standard deviations

** *P* < 0.01

*** *P* < 0.001

### Analysis of the chain mediation model

The mediating effects of perceived stress and bodily appreciation on mindfulness and life satisfaction were investigated using Hayes’ SPSS macro-PROCESS. The findings indicated that mindfulness had a significant effect on life satisfaction (*β* = 0.439, *P* < 0.001). After accounting for mediating variables, mindfulness also showed a significant direct effect on overall well-being (*β* = 0.3135, *P* < 0.001). Additionally, it was negatively correlated with perceived stress (*β* =  − 0.3077, *P* < 0.001) and positively correlated with body appreciation (*β* = 0.3519, *P* < 0.001). Life satisfaction was positively predicted by body appreciation (*β* = 0.152, *P* < 0.01), negatively predicted by perceived stress (*β* =  − 0.183, *P* < 0.01). Table 3 displays the findings.

**Table 3:** Regression analysis between variables.

**Outcome variables**	**Predictive variables**	**R-sq**	**F**	*β*	**SEs**	**t**	**LLCL**	**ULCL**
BAS (M1)	CAMSR(X)	0.123	43.817	0.351	0.111	6.619^**^	0.517	0.955
PSS(M2)	CAMSR(X)	0.201	38.948	−0.307	0.076	−5.665^**^	−0.582	−0.282
	BAS(M1)			−0.235	0.036	−4.34^**^	−0.23	−0.086
SWLS(Y)	CAMSR(X)	0.253	34.819	0.313	0.129	5.673^**^	0.478	0.987
	BAS(M1)			0.152	0.060	2.815^**^	0.051	0.289
	PSS(M2)			−0.183	0.091	−3.333^**^	−0.486	−0.125
SWLS(Y)	CAMSR(X)	0.192	73.999	0.439	0.119	8.602^**^	0.791	1.261

**Notes.**

CAMSR Mindfulness BAS Body appreciation PSS Perceived stress SWLS Life satisfaction M1 BAS M2 PSS X CAMSR Y SWLS

** *P* < 0.01.

**Figure 2:**
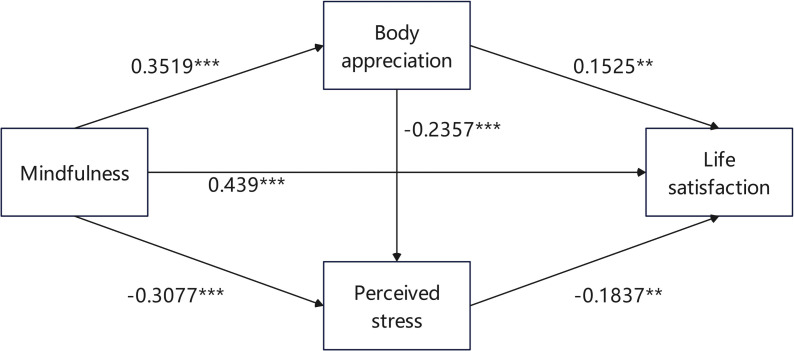
Diagram of chain mediation. ^∗∗^*p* < 0.01, ^∗∗∗^*p* < 0.001.

The mediating effect test showed that the sequential mediating effect of body appreciation and perceived stress was significant, and the bootstrap 95% CI excluded 0, indicating that body appreciation and perceived stress were mediating factors of mindfulness affecting life satisfaction. The total effect of mindfulness on life satisfaction, mediated by body appreciation and perceived stress, is 1.0266. Specifically, the mediating effect between body appreciation and stress perception is composed of indirect impacts produced by the following three pathways: (1) the indirect effect of mindfulness → body appreciation → life satisfaction pathway1 (0.1255); (2) the indirect effect of mindfulness → perceived stress → life satisfaction pathway2 (0.1322); (3) the indirect effect of mindfulness → body appreciation → perceived stress → life satisfaction pathway3 (0.0356). The results are shown in Table 4. The path of mindfulness to life satisfaction in breast cancer patients is shown in Fig. 2.

**Table 4:** Bootstrapping indirect effects and 95% confidence intervals (CI) for the mediational model.

	Effect	BootSE	BootLLCI, BootULCI
Total effect	1.0266	0.1193	0.7918, 1.2614
Direct effect	0.7332	0.1292	0.4789, 0.9875
TOTAL indirect effect	0.2934	0.0944	0.1355, 0.5036
Ind1	0.1255	0.0656	0.0111, 0.2705
Ind2	0.1322	0.0641	0.0335, 0.2851
Ind3	0.0356	0.0167	0.0084, 0.0737

**Notes.**

Ind1 mindfulness → body appreciation → life satisfaction.

Ind2 mindfulness → perceived stress → life satisfaction.

Ind3 mindfulness → body appreciation → perceived stress → life satisfaction; Confidence interval does not contain zeros.

## Discussion

This study constructed a chain mediation model involving “mindfulness, body appreciation, perceived stress, and life satisfaction” to explore the psychological mechanisms through which mindfulness influences life satisfaction. It is found that the mindfulness can directly affect not only the life satisfaction of breast cancer patients, but also indirectly affect life satisfaction through perceived stress and body appreciation.

First, this research suggested H1 by demonstrating that body appreciation mediates the influence of mindfulness on overall well-being in cancer of the breast patients. Specifically, it is shown that mindfulness indirectly enhances the well-being of individuals with breast cancer by enhancing their positive appreciation of their own bodies, and this hypothesis was also supported. According to the research results, which are in line with those of Cox AE and other researchers, the more mindful one is, the more one appreciates one’s body, and as a result, the more satisfied one is with their life (Cox & McMahon, 2019).

Fatigue, lymphedema, hair loss, and gastrointestinal symptoms resulting from breast cancer treatment can lead to negative perceptions of a woman’s own body shape and lower quality of life (Akram et al., 2017). One of the most significant patient-reported surveillance results in breast cancer, according to the ICHOM Breast Cancer Working Group, is well-being and body perception (Fahad Ullah, 2019; Maughan, Lutterbie & Ham, 2010). For example, among the 243 premenopausal individuals who have survived breast cancer, Arraras et al. (2016) found that those with a more positive body image experienced fewer instances of poor quality of life. An investigation of 406 females that had recently had breast cancer surgery revealed that people with a more positive body perception had higher overall quality of life (Ettridge et al., 2022). A greater awareness of breast cancer patients’ body image can aid in the development and improvement of treatment measures as well as optimal procedures in service delivery.

The Three Axiom models of mindfulness (Dahl & Davidson, 2019; Crane et al., 2017; Jankowski & Holas, 2014) identify three fundamental elements: intention, focus, and mindset, specifically, empathy with others and overall well-being by focusing on the present moment, observing inner and outer experiences. The initial step in utilizing mindfulness to foster a positive body image, as outlined by the Three Axioms of Mindfulness, involves embracing self-acceptance. This means resisting the sway of external opinions, breaking free from patterns of negative and self-critical thoughts, and concentrating on cultivating your own sense of positivity (Culbreth & Spratling, 2022; Giannou & Mantzios, 2023). And focus your attention on the bodily sensations and perceive the positive signals sent by the body. A randomized, single-blind, controlled trial involving 60 women aged 18–45 years who received a 16-week intervention with mindfulness-based art therapy (MBAT) found significant improvements in body appreciation, consistent with the results of this study (Bafghi et al., 2024). Persons with significant amounts of mindfulness can relate to their physicality in a more balanced way, avoid over-identifying perceived physical impairments, increase levels of body appreciation, and thus improve patient life satisfaction (Swami et al., 2020).

Secondly, this study supports Hypothesis 2 by demonstrating that perceived stress mediates the relationship between mindfulness and life satisfaction. These findings suggest that increasing mindfulness may reduce perceived stress in breast cancer patients, which could subsequently enhance their life satisfaction. Those suffering from breast cancer can become uneasy and anxious outdoors due to noticeable alterations in their looks and nervousness about inquisitive stares from other people (Kołodziejczyk & Pawłowski, 2019). Improving your level of self-mindfulness can assist control such pressures efficiently.

The theory of mindfulness holds that consciously paying attention with an open mind frees the individual from emotions, thoughts, and stories related to themselves (Davidson & Kaszniak, 2015). Mindfulness, for example, increases self-awareness, allowing people to recognize indications of stress. Research indicates that individuals with high levels of mindfulness may have an enhanced awareness of their stress levels, which can lead to the development of effective coping strategies and help alleviate the adverse impacts of stress (Lu et al., 2019; Kckaou et al., 2023).

Furthermore, it has been suggested that people with mindfulness are more capable of handling a range of stressful events, that could potentially reduce the correlation between stress and negative psychological results (Ghawadra et al., 2020). Regarding the physiological mechanism of mindfulness in relieving stress, Creswell & Lindsay (2014) suggest that mindfulness works in two ways: by increasing activity in the prefrontal cortex, which increases inhibition of stress-processing areas to reduce stress response, and by lowering levels of stress-related hormones such as adrenaline and noradrenaline. A randomized controlled trial discovered that a six-week online mindfulness help could improve self-acceptance, minimize perceived stress, and poor body perception among breast cancer patients (Chang et al., 2022). Related studies found that mindfulness is a protective factor of mental health, negatively correlated with stress, positively correlated with happiness and life satisfaction, which is consistent with the results of this study (Jayewardene et al., 2017).

Ultimately, this study suggests Hypothesis 3 by demonstrating that body appreciation and perceived stress serve as sequential mediators in the relationship between mindfulness and life satisfaction. This finding indicates that a positive body image, coupled with lower levels of perceived stress, may be fundamental to the relationship between mindfulness and life satisfaction. Allowing and accepting a bad feeling in the body and maintaining a positive attitude toward the body can reduce perceived stress and bad emotions in the face of pain and improve life satisfaction (Tao et al., 2023). The extent of mindfulness is a crucial element in mental health, especially during periods of experiencing setbacks. body appreciation is considered a protective factor for mental health (Hooper et al., 2024). When individuals accept their physical appearance, they experience a notable decrease in negative emotions and perceived stress. Additionally, an appreciation for one’s body contributes to both the prevention and encouragement of seeking help for psychological disorders (Ersoy, Aslançin & Yardı mcı, 2024). Researchers have lately demonstrated a growing interest in mindfulness’s stress-relieving effects. A number of researchers have demonstrated that therapies based on mindfulness have considerable advantages in both clinical and other settings, such as a meta-analysis that found mindfulness-based therapies reduced anxiety and depression in cancer patients (Chayadi, Baes & Kiropoulos, 2022). Mindfulness seems to raise awareness, help subjects deal with stressful situations, and reduce anxiety, depression, and stress symptoms. In a similar vein, the present chain-mediated model uncovers the mechanism through which mindfulness influences life satisfaction among breast cancer patients. Therefore, positive body appreciation and lower levels of perceived stress can improve life satisfaction.

The future direction of cancer treatment is moving towards “precision, individualization and long-term management”, and the complexity of treatment, long-term health management stress, and uncertainty anxiety about recurrence may profoundly affect patients ’physical and mental health and quality of life (Sonkin, Thomas & Teicher, 2024). Mindfulness, as a scientifically proven practice, has unique advantages in stress management. Therefore, structured mindfulness interventions (*e.g.*, MBSR, MBCT) are incorporated into supportive care for breast cancer patients to guide patients to actively participate and improve quality of life while pursuing longer survival.

## Practical implications

The findings of this study have major consequences in theory as well as practice for enhancing life satisfaction of breast cancer patients. At a theoretical level, this research validated the relationship between mindfulness, body appreciation, perceived stress, and life satisfaction, while also uncovering the internal mechanisms through which mindfulness affects life satisfaction *via* a serial mediation model. Specifically, mindfulness directly enhances life satisfaction and indirectly impacts it by fostering greater body appreciation and reducing perceived stress. This finding supports key viewpoints in attention control theory and cognitive-behavioral models, which posit that mindfulness improves mental health by altering individuals’ responses to negative events and reducing negative cognitions and emotional responses (Morgan, 2003). First, attention control theory posits that mindfulness enhances individuals’ ability to manage their attention on present experiences, thereby diminishing distractions and emotional overreactions, while fostering greater awareness and acceptance of the present moment (Jankauskiene et al., 2024). Secondly, the cognitive-behavioral model emphasizes that mindfulness changes the way individuals respond to negative emotions and thoughts, reducing perceived stress and thereby improving mental health (Butler et al., 2006). A randomized controlled trial found that mindfulness-based stress management programs improved sleep and stress status in cancer patients better than cognitive behavioral therapy alone (Garland et al., 2014). Furthermore, previous studies have concentrated on the negative body perception experienced by individuals with breast cancer, exploring whether the disease leads to a detrimental attitude toward their bodies. In contrast, this research investigates the positive body image among breast cancer patients.

The findings of this research indicate that mindfulness practice may substantially boost the level of life satisfaction among breast cancer patients. Therefore, at a practical level, hospitals and communities can guide breast cancer patients to conduct mindfulness training and help breast cancer patients enhance their consciousness in the current time and reduce stress using techniques like mindfulness meditation, mindful breathing, and yoga. In addition, during mindfulness intervention, attention should also be paid to the body appreciation attitude and pressure felt by breast cancer patients. By enhancing their physical connection with nature, individuals can experience a sense of respect for their bodies, elevate their appreciation for themselves, and alleviate stress. Finally, the results support the effectiveness of the chain mediation model, suggesting that mindfulness can improve life satisfaction by improving body appreciation and reducing perceived stress. Consequently, psychological intervention strategies must consider these mediating factors and aim to substantially improve the quality of life for individuals with breast cancer by implementing interventions like meditation training.

## Limitations and Future Directions

This research has a few drawbacks. Since this was a cross-sectional survey, it is crucial to recognize that causal relationships between variables cannot be inferred. Instead, only exploratory analysis can be conducted, with any findings requiring validation through subsequent intervention studies. In future work, the longitudinal follow-up study method may be employed to facilitate in-depth discussions on the questions raised in this investigation, enhancing the understanding of the correlation among mindfulness, body appreciation, perceived stress, and life satisfaction. Furthermore, potential bias may impact the methodology of the questionnaire, potentially compromising the study’s validity if respondents do not answer truthfully. Subsequent research endeavors should enhance this field by incorporating experimental and qualitative approaches. Additionally, the study’s sample size was limited to 312 participants from a single region, thereby constraining the generalizability of the findings.

##  Supplemental Information

10.7717/peerj.20485/supp-1 Supplemental Information 1 Raw data View using SPSS software. The specific meanings of the numbers can be found in the data view.

## Additional Information and Declarations

### Data Availability

The following information was supplied regarding data availability: The raw measurements are available in the Supplementary File.
